# Daily Vaginal Microbiota Fluctuations Associated with Natural Hormonal Cycle, Contraceptives, Diet, and Exercise

**DOI:** 10.1128/mSphere.00593-20

**Published:** 2020-07-08

**Authors:** Stephanie D. Song, Kalpana D. Acharya, Jade E. Zhu, Christen M. Deveney, Marina R. S. Walther-Antonio, Marc J. Tetel, Nicholas Chia

**Affiliations:** a Microbiome Program, Center for Individualized Medicine, Mayo Clinic, Rochester, Minnesota, USA; b Neuroscience Department, Wellesley College, Wellesley, Massachusetts, USA; c Department of Psychology, Wellesley College, Wellesley, Massachusetts, USA; d Division of Surgical Research, Department of Surgery, Mayo Clinic, Rochester, Minnesota, USA; e Division of Gynecologic Research, Department of Obstetrics and Gynecology, Mayo Clinic, Rochester, Minnesota, USA; University of Michigan-Ann Arbor

**Keywords:** estrogens, lactobacillus, menstrual cycle, microbiome, progesterone, time-longitudinal analysis, women’s health

## Abstract

The microorganisms of the vaginal tract are critical for vaginal and reproductive health. However, the regulation of these microorganisms is not well understood. Therefore, we investigated whether different factors regulate the vaginal microbiota of healthy college-aged women (*n *=* *26) with high temporal resolution by collecting daily self-administered vaginal swabs and using 16S rRNA sequencing for bacterial identification. As expected, vaginal microbiota clustered into five predefined community state types. Vaginal microbial diversity, stability, and *Lactobacillus* abundances were associated with the menstrual cycle and hormonal contraceptive use. Vaginal microbial diversity, as measured using the Shannon index, increased during menses (*P* < 0.001), while *Lactobacillus* abundances decreased (*P* = 0.01). The covariance of these microbial measures with previously established estradiol levels suggests that estrogens can regulate vaginal microbiota. Moreover, the use of hormonal contraceptives may alter the temporal dynamics of the vaginal microbiota and decrease *Lactobacillus* abundances, depending on hormonal content and release method. Interestingly, intrasample diversity was greater in participants on a vegetarian diet (*P* = 0.004) and among participants who exercised more (*P* = 0.04). These findings indicate that ovarian hormones, diet, and exercise can regulate vaginal microbial composition and stability and may impact vaginal and reproductive health.

**IMPORTANCE** The vaginal microbiome is a critical component of women’s sexual and reproductive health, with variations in microbial composition, particularly the loss of *Lactobacillus* species, being implicated in gynecologic and obstetric diseases. Given that the vaginal microbiome is so crucial, why do vaginal microbial profiles vary strikingly from person to person and even change over time within the same person? In the present study, which tracked the daily vaginal microbiomes of young healthy women through different lifestyles, we found that use of a locally released progestin contraceptive, a vegetarian diet, and intense exercise appear to lead to vaginal microbiome alterations and loss of *Lactobacillus* species. The impact of these vaginal microbiome changes on immediate and long-term health remain to be investigated.

## Abstract (precis)

The vaginal microbiome is a critical component of women’s sexual and reproductive health, with variations in microbial composition, particularly the loss of *Lactobacillus* species, being implicated in gynecologic and obstetric diseases. Given that the vaginal microbiome is so crucial, why do vaginal microbial profiles vary strikingly from person to person and even change over time within the same person? In the present study, which tracked the daily vaginal microbiomes of young healthy women through different lifestyles, we found that use of a locally released progestin contraceptive, a vegetarian diet, and intense exercise appear to lead to vaginal microbiome alterations and loss of *Lactobacillus* species. The impact of these vaginal microbiome changes on immediate and long-term health remain to be investigated.

## INTRODUCTION

The microorganisms of the vaginal tract are critical for vaginal and reproductive health. *Lactobacillus* spp. are a major component of most vaginal microbial communities (1–3) and key contributors to the defense mechanisms provided by those communities (4–10). Beginning from puberty until menopause, vaginal microbial communities are commonly dominated by a single *Lactobacillus* species (1, 11–13). Moreover, *Lactobacillus* abundance increases markedly during pregnancy (14, 15). *Lactobacillus* is proposed to protect against infections (4) and maintain a healthy vaginal epithelium (16) through production of lactic acid (17), which creates a low-pH vaginal environment unsuitable for pathogenic bacterial growth (8, 9). Lack of *Lactobacillus* and high alpha diversity, along with a high vaginal pH, can be key clinical characteristics of bacterial vaginosis (BV) (10, 18, 19). BV and vaginal microbial dysbiosis are linked to preterm birth (20–22), increased risk of HIV transmission (23–26), increased risk of HPV infection, and cervical, endometrial, and ovarian cancers (6, 27–29). However, some women (12) and transgender men (30) with low *Lactobacillus* and high diversity present as asymptomatic and otherwise healthy.

Despite the dominance of *Lactobacillus* in most vaginal microbiomes, the composition of any given vaginal microbiome is highly individualized. Moreover, vaginal microbial profiles are dynamic and may change over time (31), even transitioning between two community types within a few days (32). Therefore, it is important to understand the host-microbiome interactions that regulate the vaginal microbiota and drive such individualization. Several host factors that influence the vaginal microbiota have been identified, including menses (31–37), intercourse (38, 39), and hormonal contraceptive use (38, 40–42). However, the exact nature and mechanism of how these components regulate vaginal microbiota remain unknown. For instance, there are conflicting accounts on how menses alters the vaginal microbiome. Some reports found decreases in L. crispatus and increases in BV-associated bacteria during menses (35, 37). In contrast, another study found no change in alpha diversity during menses, despite a decrease in stability (32). It is also unclear whether the menstrual changes indicate a relationship between vaginal microbiota and estrogen levels (31, 32, 38, 40–43) or other factors such as progesterone levels (44) or the presence of menstrual blood (16). Whether diet, exercise, or even mood interact with vaginal microbiota is less understood, even though the influences of these host factors have been studied extensively in other microbial systems, including the gut microbiome (45–47).

Assessing the relationships between the vaginal microbiome and its modulatory factors has been difficult due to a need for high temporal resolution to capture rapid changes occurring over a few days (32, 37). Previous longitudinal vaginal microbial studies with weekly (48), biweekly (32), or monthly (34, 38) samples may not have captured such changes. Therefore, this study investigated how the vaginal microbiome changes over the menstrual cycle and potential relationships with contraceptive use, diet, exercise, and mood at high temporal resolution. These findings reveal increased vaginal microbial diversity and decreased *Lactobacillus* relative abundances during menses, suggesting that vaginal microbial composition cycles with the menstrual cycle. In addition, altered vaginal microbial profiles were associated with progestin-only contraceptive use, a vegetarian diet, and intense exercise.

## RESULTS

To characterize the vaginal microbiome at high temporal resolution, volunteers from Wellesley College (ages 18 to 22 years) submitted daily vaginal swab samples for 10 weeks while recording menstrual status, contraceptive use, diet, exercise, and mood. All procedures involving participants were approved by the Wellesley College Institutional Review Board (IRB), and written informed consent was obtained from all participants. Of 36 initially enrolled participants, 26 (72%) submitted 12 or more samples during the study and were thus included in the analysis. Sample collection and sequencing were performed across 2 years (*n *=* *14 in year 1; *n *=* *12 in year 2) in two separate batches. Four individuals, or repeat participants, participated in both years of the study. Sample sizes consider data from both years of each repeat participant as *n *=* *2, unless stated to be “unique” participants, in which case each repeat participant is *n *=* *1. Bacteria present in each sample were identified using high-throughput 16S rRNA gene sequencing.

### Community state types of vaginal microbiota.

**FIG 1:**
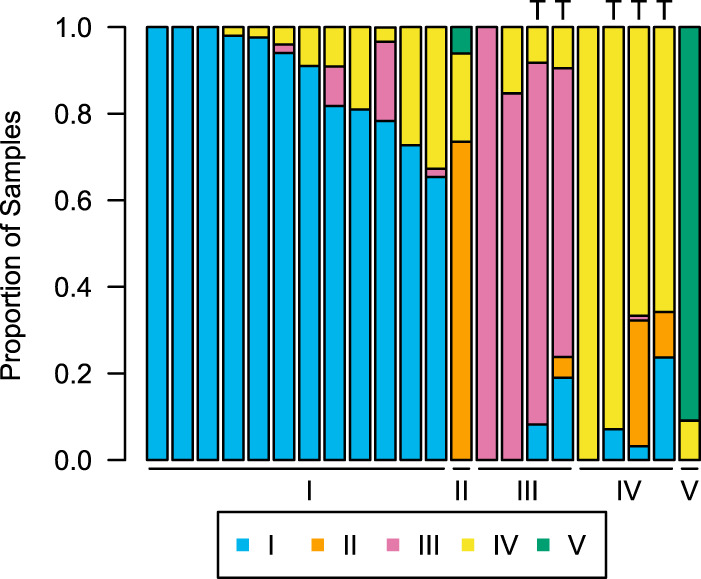
Samples cluster into five main community state types (CSTs). Data for 22 unique participants are shown, with each individual’s proportion of samples that belong to types I (L. crispatus dominance), II (L. gasseri), III (*L. iners*), IV (diverse), and V (L. jensenii). Participants who displayed long-term transitions from one CST to another during the study are indicated by “T.”

**FIG 2:**
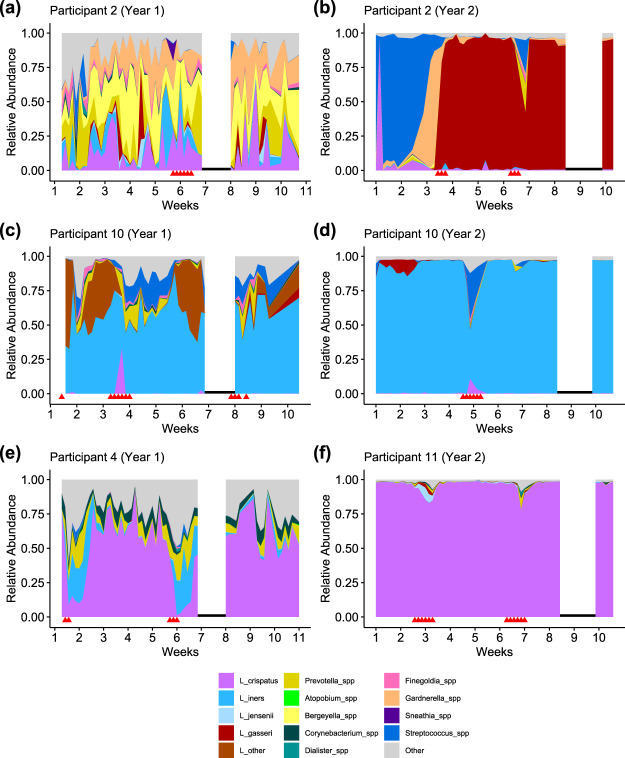
Examples of participant phylotypes. (a and b) Participant 2, who was enrolled in both years of the study, was type IV (diversity) in year 1 (a) and transitioned to type II (L. gasseri) in year 2 between weeks 3 to 4 (b). (c and d) Participant 10, who was also enrolled in both years, was type III (*L. iners*). (e and f) Participant 4 (e) was type I (L. crispatus) with relatively higher diversity, while participant 11 (f) was also type I with low diversity. Red triangles indicate days on which the participant reported menstruation. Black lines indicate the college’s spring break, when samples were not collected.

We confirmed previous characterizations of the vaginal microbiota (12), which described the vaginal microbial communities of reproductive-age women as individualized but clustering into five community state types (CST). Types I, II, III, and V are dominated by Lactobacillus crispatus, L. gasseri, L. iners, and L. jensenii, respectively, while type IV is more diverse with higher abundances of anaerobic bacteria. We found concordance in our cohort; communities were highly individualized (see Fig. S1 in the supplemental material) and mainly clustered into the five predefined CST (Fig. 1). Of 22 unique participants, a majority (>50%) of samples from 12 participants were type I (Fig. 2e and f), a majority of samples from 4 participants each were types III (Fig. 2c and d) and IV (Fig. 2a), and a majority of samples from 1 participant each were types II and V.

10.1128/mSphere.00593-20.2 FIG S1 Principal component analysis (PCoA) reveals that vaginal microbial communities are highly individualized, and composition differs during menses. PCoA (Bray-Curtis) of vaginal communities are colored by individual (A) or by menstruation status (red = menses) (B). Permutational analysis of variance (PERMANOVA) reveals significant differences in composition by individual (*P = *0.001) and by menstruation status (*P = *0.001). Circles are year 1 samples; triangles are year 2 samples. Download FIG S1, EPS file, 0.7 MB. Copyright © 2020 Song et al. 2020 Song et al. This content is distributed under the terms of the Creative Commons Attribution 4.0 International license.

Five participants displayed long-term transitions (Fig. 1 and Fig. 2b), shifting from one CST to another and persisting in the latter state for 10 days or more. Because previous findings suggest that menses is associated with decreased community stability and transient CST transitions (32, 34), we examined whether long-term transitions were also associated with menses. Given that all five of the participants who transitioned did so during menses, a binomial test indicates that long-term transitions were in fact more likely to occur during menses (*P* < 0.001).

### Menstrual fluctuations of vaginal microbiota.

**FIG 3:**
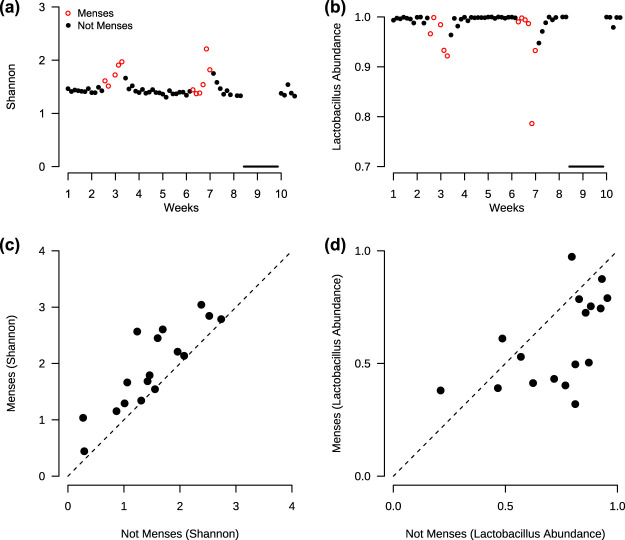
Diversity (Shannon index) increases and *Lactobacillus* decreases during menses. (a and b) Example of individual (participant 11) with increase in diversity (a) and decrease in *Lactobacillus* (b) during menses. Black line indicates the college’s spring break, when samples were not collected. (c) Across all participants (*n *=* *17), the average diversity is greater during menses compared to not menses (Wilcoxon signed rank, *P* < 0.001). (d) The average *Lactobacillus* relative abundance is lower during menses compared to not menses (Wilcoxon signed rank, *P = *0.01).

To investigate the relationship between vaginal microbial composition and the menstrual cycle, we examined the vaginal microbiota of participants who reported menstruating (*n *=* *17, including 3 repeat participants). Alpha diversity increased during menses as measured using the Shannon index (Wilcoxon signed rank, *P < *0.001; Fig. 3a and c). Beta diversity (Bray-Curtis) analysis revealed compositional differences between menstrual statuses (PERMANOVA [permutational multivariate analysis of variance], *P* = 0.001; Fig. S1). At the genus level, *Lactobacillus* relative abundances decreased during menses (Wilcoxon signed rank, *P* = 0.01; Fig. 3b and d). However, no universal increases or decreases during menses were found at the species level (Fig. S2). Despite the lack of a single species that changed significantly, some overall trends emerged. *Lactobacillus* species tended to decrease in abundance during menses, in accordance with our previous finding, while the species that tended to increase in abundance during menses, such as *Streptococcus* spp., *Peptostreptococcus* spp., and *Anaerococcus* spp., were species that are often cultured on blood agar (49–51). Taken together, these results reveal that alpha diversity increases during menses, concurrent with a decrease in *Lactobacillus* abundances at the genus level, though compositional changes are individualized at the species level.

10.1128/mSphere.00593-20.3 FIG S2 No generalized shifts occur during menses at the species level. (A) Volcano plot of species with unadjusted *P* < 0.05 (Wilcoxon signed rank) and fold change in relative abundance of <0.5 or >2. (B) Difference in cube root relative abundance during menses in 14 identified species, for each participant. No species remained significant after *P* value adjustment for the false discovery rate (Benjamini-Hochberg). Cube root difference rather than fold change was used to capture instances when a species was not present during either menses or not menses in a participant. Species are labeled 1 to 14 in both plots. Download FIG S2, EPS file, 1.3 MB. Copyright © 2020 Song et al. 2020 Song et al. This content is distributed under the terms of the Creative Commons Attribution 4.0 International license.

Next, we investigated the temporal dynamics of the vaginal microbiota to assess the hypothesis that the community varies over the menstrual cycle, and that these variations relate to cycling estrogens. Samples from regularly cycling participants with at least two cycles’ data (*n *=* *8) were normalized to 28 days. The potential effect of exogenous hormones on the vaginal microbiota was accounted for by separating participants by contraceptive use into three groups: no contraceptives (*n *=* *4); estrogen and progestin combined, systemic release contraceptives (C-Systemic, *n *=* *2); and progestin-only, local release contraceptives (P-Local, *n *=* *2). All the C-Systemic contraceptives involved a hormone-free week, while the P-Local contraceptives did not. Log Jensen-Shannon divergence rate of change (32) was used to measure the “stability” or “rate of change” of a vaginal microbial community over time. Alpha diversity (Shannon index), community rate of change (log Jensen-Shannon), and the relative abundance of *Lactobacillus* were modeled over the normalized menstrual cycle using local regression (loess).

**FIG 4:**
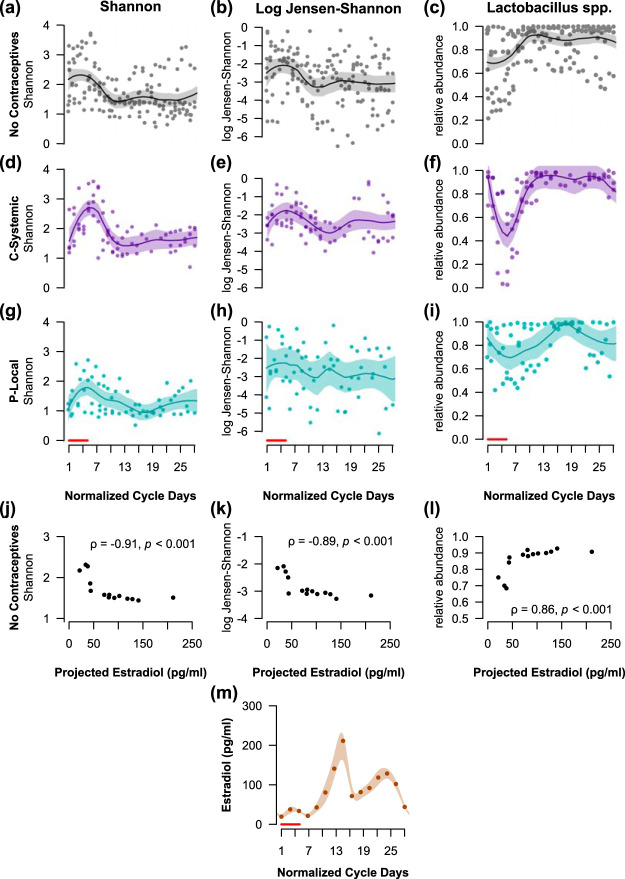
Vaginal microbial diversity, stability, and *Lactobacillus* correlate with previously established estradiol levels (52) across the menstrual cycle. The Shannon index (a, d, and g) and log Jensen-Shannon rate of change (b, e, and h) increase during menses, with a concurrent decrease in *Lactobacillus* relative abundance (c, f, and i). Participants not using hormonal contraceptives (a to c, *n *=* *4), using estrogen and progestin combined, systemically released contraceptives (d to f, *n *=* *2), or using progestin-only, locally released contraceptives (g to i, *n *=* *2) are shown. The local regression (loess) estimates of diversity, stability, and *Lactobacillus* correlate (Spearman correlation) with estradiol values transformed from Minassian and Wu (52) (m) for participants not using hormonal contraceptives (j to l). Spearman correlations (ρ) and *P* values are displayed in panels j to l. The shaded regions in panels a to i and panel m are 95% confidence bands of the local regression fit. Red lines indicate the first 5 days when menses typically occurs.

Consistent with our present and others’ previous findings (32), there were increases in alpha diversity and rate of change, with a corresponding decrease in *Lactobacillus* during the first 5 days of the menstrual cycle, when menses typically occurs. The community changes were not fully explained by changes in migration rates, the rates of new species being introduced into the community and extinction of existing community members (Fig. S3). Therefore, the associated changes with menses reflect alterations in community evenness and structure. To further assess whether the vaginal microbiota covaries with estradiol levels, the regression estimates of alpha diversity, rate of change, and *Lactobacillus* abundance were correlated with previously established estradiol values adapted from Minassian and Wu (52), while acknowledging the limitation that these projected estradiol levels are only estimates of actual estradiol levels. Among participants not using hormonal contraceptives and presumably following a natural cycling of ovarian hormones (Fig. 4a to c), all three microbial measures correlated significantly (Spearman correlation, *P < *0.001; Fig. 4j to l) with the projected estradiol levels (Fig. 4m) across the menstrual cycle. Interestingly, the participants using C-Systemic contraceptives (Fig. 4d to f) showed similar periodic fluctuations of alpha diversity, community rate of change, and *Lactobacillus* abundance to those of the no contraceptive group, while participants using P-Local contraceptives did not follow this distinct pattern (Fig. 4g to i). Given that hormonal contraceptives alter the cyclic release of hormones in women, estradiol levels in participants using contraceptives (Fig. 4d to i) were not compared to those of cycling women from Minassian and Wu (52). To quantify these periodic fluctuations, we estimated the power and frequency of these periodic patterns using least-squares spectral analysis (LSSA) (53), an extension of the Fourier method to unevenly spaced data. While the vaginal microbial communities of both the no-contraceptive and the C-Systemic contraceptive groups closely followed 14- and 28-day periods, the P-Local contraceptive group did not (Fig. S4).

10.1128/mSphere.00593-20.4 FIG S3 Changes in migration rates do not fully explain diversity changes across the menstrual cycle. Introduction, defined as the number of OTUs present on a given normalized cycle day but not present the previous collection day, and extinction, defined as the number of OTUs present the previous collection day but not present on the given day, are shown for all participants (*n *=* *17). Migration rates were modeled using loess (span = 0.5). No dependence on cycle days was found for OTU introduction (ANOVA, *P* = 0.1) or extinction (ANOVA, *P* = 0.3) Red lines indicate the first five days when menses typically occurs. Download FIG S3, EPS file, 0.4 MB. Copyright © 2020 Song et al. 2020 Song et al. This content is distributed under the terms of the Creative Commons Attribution 4.0 International license.

10.1128/mSphere.00593-20.5 FIG S4 Least-squares spectral analysis (LSSA) of alpha diversity (Shannon index), community rate of change (log Jensen-Shannon), and *Lactobacillus* relative abundance over the normalized menstrual cycle. Fluctuations following approximately 28-day and 14-day periods for participants not using hormonal contraceptives (*n *=* *4, top row) and those using combined, systemic release (C-Systemic) contraceptives (*n *=* *2, middle row) but not for those using progestin-only, local release (P-Local) contraceptives (*n *=* *2, bottom row). Least-squares spectral analysis (LSSA, R package “nlts” function “spec.lomb”), an extension of the Fourier analysis to unevenly spaced data, was used to calculate the frequency and amplitude of periodic community changes normalized to a 28-day cycle. Periods (days) of major peaks are labeled. Download FIG S4, EPS file, 1.1 MB. Copyright © 2020 Song et al. 2020 Song et al. This content is distributed under the terms of the Creative Commons Attribution 4.0 International license.

### Hormonal contraceptives alter *Lactobacillus* dominance.

**FIG 5:**
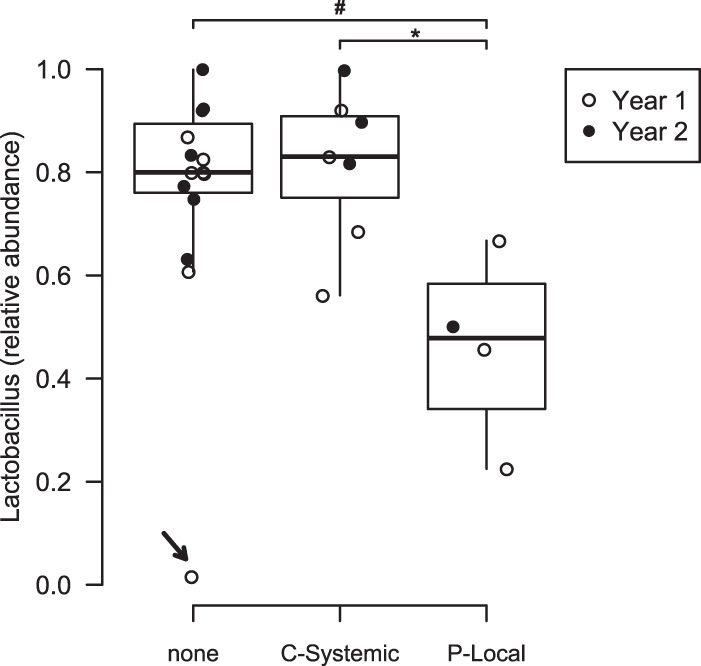
Average *Lactobacillus* relative abundances differ by contraceptive use. Data are shown for participants using no hormonal contraceptives (none, *n *=* *12); combined, systemic release contraceptives (C-Systemic, *n *=* *7); or progestin-only, local release contraceptives (P-Local, *n *=* *4). Participants using P-Local contraceptives have significantly less *Lactobacillus* than those using C-Systemic contraceptives (Tukey HSD, *P = *0.04) and trending less than those using no contraceptives (Tukey HSD, *P = *0.07). These differences become significant (*P* < 0.01) after removal of the outlier participant (arrow). Open circles, year 1 samples; closed circles, year 2 samples. *, *P < *0.05; #, 0.05 < *P < *0.1.

If the vaginal microbiota is influenced by estrogens or progestins, we expect that hormonal contraceptive use would cause a generalized shift in the vaginal community. Therefore, we included all 23 participants in a cross-sectional examination of the average relative abundances of *Lactobacillus* by contraceptive use. It should be noted that three participants were excluded from the full cohort of 26 because each used a unique form of contraceptive, and disclosure of this information would compromise anonymity. After adjusting for year of the study, participants not using hormonal contraceptives (*n *=* *12) and those using C-Systemic contraceptives (*n *=* *7) presented high average *Lactobacillus* abundances. Meanwhile, those using P-Local contraceptives (*n *=* *4) presented lower average *Lactobacillus* abundances (Fig. 5). In summary, we found that participants not using hormonal contraceptives and those using combined contraceptives display similar periodic fluctuations of vaginal microbiota that correspond to stages of the menstrual cycle, and high average *Lactobacillus* abundances. However, participants using progestin-only contraceptives had altered periodic fluctuations of vaginal microbiota and low average abundances of *Lactobacillus*.

### Relationships with vegetarian diet, intense exercise, and mood.

**FIG 6:**
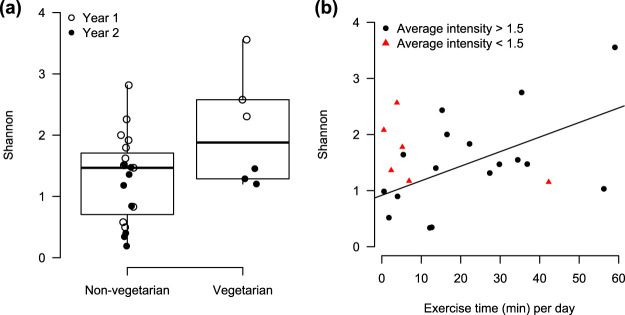
Alpha diversity is associated with diet and exercise. (a) Average Shannon index was found to be higher among participants following a vegetarian diet (*n *=* *6) compared to a nonvegetarian diet (*n* = 19; sanon, *P* = 0.004). (b) Average Shannon index increases with exercise time (minutes) per day. However, this effect was only among participants with an average exercise intensity of >1.5 (*n *=* *17), where 1 = low, 2 = moderate, and 3 = high (linear regression, β = 0.026, *P* = 0.04, *R^2^* = 0.29).

We investigated potential relationships between the vaginal microbiota and long-term dietary patterns, exercise time and intensity, and mood. Participants following a vegetarian diet (*n *=* *6) exhibited higher average vaginal microbial diversity than nonvegetarians (*n *=* *19; *P* = 0.004; Fig. 6a), after adjusting for year using stratified analysis with nonparametric covariable adjustment (sanon) (54). Meanwhile, no significant relationships were found between the vaginal microbiota and specific nutrient intake, including sugar, fiber, protein, or fat (see Fig. S5 in the supplemental material).

10.1128/mSphere.00593-20.6 FIG S5 Alpha diversity is not significantly correlated with average nutrient intake. No significant relationships were found between average Shannon index and average sugar, fiber, fat, or protein intake, as a proportion of total daily caloric intake (linear regression, *P > *0.1). Download FIG S5, EPS file, 0.4 MB. Copyright © 2020 Song et al. 2020 Song et al. This content is distributed under the terms of the Creative Commons Attribution 4.0 International license.

The average alpha diversity was observed to increase with average exercise time-per-day (linear regression, *P = *0.02; Fig. 6b), calculated as the total number of minutes of exercise reported over the number of study days. However, the positive correlation was only among individuals who self-reported an average exercise intensity above 1.5 (*n *=* *17; exercise intensity: 1, low; 2, medium; 3, high). The results remained after adjusting for year (Fig. S6). No significant relationship was found between the vaginal microbiota and affect or arousal ratings (Fig. S7). This lack of an association may be due to the low number of participants (*n *=* *5) who consistently reported these measures, since this aspect of the study was only implemented in year 2.

10.1128/mSphere.00593-20.7 FIG S6 Alpha diversity increases with exercise time (minutes) per day after adjusting for batch effects by year. Only participants with an average self-reported exercise intensity greater than 1.5 (*n *=* *17; 1 = low, 2 = moderate, 3 = high) were included (circles). Linear regression was used with average Shannon index as the outcome, and average exercise time per day, study year, and the interaction as the predictors (exercise time per day, β = 0.021, *P* = 0.08; year 2, β = −0.764, *P* = 0.26; exercise time per day × year 2, β = 0.005, *P* = 0.87; *R^2^* = 0.44). The dotted line is the fitted model for year 1 participants (open points), and the solid line is the fitted model for year 2 participants (closed points). Download FIG S6, EPS file, 0.3 MB. Copyright © 2020 Song et al. 2020 Song et al. This content is distributed under the terms of the Creative Commons Attribution 4.0 International license.

10.1128/mSphere.00593-20.8 FIG S7 Alpha diversity compared to happiness ratings or anxiety ratings within each participant. Participants were asked to self-report happiness ratings (0 = sad, 100 = happy) and arousal ratings (0 = tense, 100 = relaxed). Anxiety ratings were calculated by combining these two measures (0 = low anxiety [happy and relaxed], 100 = high anxiety [sad and tense]). Results were not significant (linear regression, *P* > 0.2). Red triangles indicate days the participant reported menstruating. Download FIG S7, EPS file, 0.8 MB. Copyright © 2020 Song et al. 2020 Song et al. This content is distributed under the terms of the Creative Commons Attribution 4.0 International license.

## DISCUSSION

The present findings reveal, with daily temporal resolution, that the vaginal microbiota in young women correlates with cycling estradiol levels, may be altered in the presence of local progestins, and is associated with dietary patterns and intense exercise. During menses, vaginal microbial diversity increases, with a concurrent decrease in *Lactobacillus* and increase in community rate of change. Alpha diversity, stability, and *Lactobacillus* abundances in individuals not using hormonal contraceptives correlate with predicted estradiol levels across the menstrual cycle. The present findings that vaginal community stability covaries with estradiol levels are consistent with those of Gajer et al. (32). However, while we found differences in alpha diversity across the menstrual cycle, Gajer et al. did not. This discrepancy in results may be due to the higher temporal resolution (daily rather than twice a week), different cohort demographics (college students 18 to 22 years old compared to participants 18 to 40+ years old), and/or greater average sequencing depth of the present study compared to the previous one (32). The present findings suggest that the exogenous hormones in contraceptives may affect the vaginal microbiota, depending on the contraceptive’s hormonal composition, release method, or the presence or absence of a hormone-free period. Furthermore, the present findings suggest that progestin-only, local release contraceptives without a hormone-free period disrupt the usual periodic fluctuations in vaginal microbiota and suppress *Lactobacillus* growth. However, the data are not sufficient to determine which characteristics of the hormonal contraceptives contributed to these differential results. In addition, these findings are limited by the small number of participants who reported at least one full cycle of menstrual data (*n *=* *8). Nonetheless, these findings support the concept that estrogens and progestins regulate the vaginal microbiota and, moreover, that local release of progestins affects *Lactobacillus* species.

Consistent with the present results, a recent meta-analysis supports the proposal that *Lactobacillus* growth in the vaginal environment is hormone-driven (31). While many reports have focused on how estrogens may promote *Lactobacillus* growth, less attention has been given to the possible role of progestins (32, 38, 40–43). The focus directed toward estrogens as promoters of *Lactobacillus* growth is based on epidemiologic observations of the major vaginal microbial changes that occur throughout the course of life. *Lactobacillus* abundances increase during puberty (55) when estrogen levels also increase (56). *Lactobacillus* markedly dominates the vaginal environment during pregnancy, also when estrogen levels are high (14, 15). Whereas *Lactobacillus* declines postmenopause when estrogens decrease, estrogen replacement therapy restores *Lactobacillus* levels (5, 57, 58). Though transgender men using testosterone exhibit lower *Lactobacillus* abundances, those using an estrogen ring have restored *Lactobacillus* levels (30). A commonly proposed mechanism by which estrogens may increase *Lactobacillus* abundances is through increasing free glycogen availability in the vaginal mucosa, which in turn supports *Lactobacillus* growth (44, 48, 59–61). However, a clear link between estrogen and free glycogen levels has proven difficult to detect. Several studies examining serum or salivary estrogen levels in comparison to glycogen and *Lactobacillus* found no associations (44, 48, 62). However, one study found progesterone to be negatively associated with glycogen levels (44). This finding, taken together with the present results, suggests that the potential effect of progestins on the vaginal microbiota is currently underestimated. Future investigations measuring endogenous and exogenous estrogens and progestins at high temporal resolution are needed for a more complete understanding of the relationship between vaginal microbial composition and sex hormones.

It is important to note that the presence of menstrual blood in the environment may contribute to the changes observed. A number of factors could contribute to this potential effect of menstrual blood, including the presence of iron, increased pH, or tampon usage during menstruation, all of which were not recorded in this study. In addition, sexual activity impacts the vaginal microbiome (38, 39), and it is likely that sexual activity patterns differ depending on menstrual status. Unfortunately, participants’ reports of sexual activity in the present study, which was an optional entry in the initial study design, could not be exported or analyzed due to a technical error in the mobile application.

Interestingly, we found that vaginal microbial diversity is higher among vegetarians than nonvegetarians, and alpha diversity increases with average exercise time per day. These results reveal the possibility that long-term diet and energy metabolism influence the vaginal microbiome. In support, studies reveal that obesity and diets with high fat, high glycemic load/energy density, and low vitamins A, C, and E and β-carotene are associated with increased risk of bacterial vaginosis (63–66). One proposed explanation for why humans are the only mammals with *Lactobacillus* dominance is that the high starch content of human diets leads to high levels of glycogen in the vaginal tract, creating a suitable environment for *Lactobacillus* (67). It will be important for future studies to investigate the impact of diet and energy metabolism on the vaginal microbiota and potential implications for vaginal health.

Lastly, no significant associations were found between vaginal microbiota and mood. Because the mood aspect was only implemented in year 2 of the study, there was a low number of participants who consistently reported mood (*n *=* *5). Therefore, the generalizability of these data is limited due to the small sample size, though the preliminary associations reported in the supplemental materials may serve as pilot data for future investigations into the effects of mood on the vaginal microbiome.

The results of this study motivate future investigations of how estrogens, progestins, diet, and exercise influence the vaginal environment. There is also a need to understand why some community state transitions are transient, while others become permanent. In addition, the present study highlights the power and importance of longitudinal studies, especially at high temporal resolution, to detect critical patterns in vaginal microbial data. The effort to understand the vaginal microbiome will ultimately facilitate discovery of new risk factors, diagnostic techniques, preventative measures, and treatments for vaginal microbial dysbiosis and other disorders related to women’s health, including ovarian and endometrial cancer.

## MATERIALS AND METHODS

### Study design.

Female students at Wellesley College (ages 18 to 22 years) participated in the study in 2017 (year 1 [Y1]) and 2018 (year 2 [Y2]) (total *n* = 32 enrolled, including 4 “repeat” participants who enrolled both years). All procedures involving participants were approved by the Institutional Review Board of Wellesley College, and written informed consent was obtained from all participants. Given methodological differences between the two collection years (see the supplemental materials and methods (Text S1]), it was not possible to combine data between years for the four repeat participants, therefore, independence between Y1 and Y2 results was assumed for repeat participants. Participants collected vaginal swab samples daily for 10 weeks, excluding the college’s spring break (lasting 1 week) during both years. 51 and 34 average swabs per participant were collected in Y1 and Y2, respectively (Fig. S8). Using a web application developed for this study, participants submitted the QR code associated with their daily swab sample, and recorded menstruation status, daily diet, exercise, and mood (Y2 only). Participants were able to report other information, including exercise (type, time, and intensity), health (wellness and weight), sexual activity (type, gender, partner number, and contraceptive used), and medication, though these data reports were optional following IRB guidelines to protect sensitive participant information. Unfortunately, a technical error occurred in exporting the sexual activity information from the mobile application and prevented the analysis of these data. Diet data were linked to nutritional information provided by the dining hall food provider, AVI Fresh. The low number of participants in the study, combined with the small student body size of the college, raised concerns of self-identification. To alleviate these concerns and maximize volunteer retention, demographic data of ethnicity and age were not collected. Details of certain data that were collected could also lead to self-identification, such as specific contraceptive types. These specific details are not reported here to provide as much privacy as possible, but the information was made available to the authors to investigate correlations or confounders based on these factors.

10.1128/mSphere.00593-20.1 TEXT S1 Supplemental materials and methods. Download Text S1, DOCX file, 0.1 MB. Copyright © 2020 Song et al. 2020 Song et al. This content is distributed under the terms of the Creative Commons Attribution 4.0 International license.

10.1128/mSphere.00593-20.9 FIG S8 Sampling frequency of study participants. Rows are individual participants, dots represent days on which a vaginal swab sample submitted, and black lines indicate the college’s spring break. Averages of 51 and 34 swabs per participant were collected in Y1 and Y2, respectively. Download FIG S8, EPS file, 0.2 MB. Copyright © 2020 Song et al. 2020 Song et al. This content is distributed under the terms of the Creative Commons Attribution 4.0 International license.

### Sample collection and storage.

Participants were provided with sterile polyester swabs (Puritan Medical Products, Guilford, ME), Falcon 15-ml conical tubes (Fisher Scientific, Hampton, NH), QR code stickers, and collection instructions. Participants were instructed to collect a pair of vaginal swab samples each morning, place samples inside the Falcon tube with a QR code label placed on the outside of the tube, scan the QR label using the web application, and store the samples at 4°C in refrigerators located in the residence halls. Samples were collected by volunteers at 12 p.m. daily on weekdays and 3 p.m. on weekends and then stored at –80°C to prevent degradation until further processing.

### Sequencing and processing.

At the conclusion of the study, samples were sent to the University of Minnesota Genomics Center (UMNGC; Y1 samples) or the Mayo Clinic (Y2 samples) for genomic DNA extraction. Immediately prior to DNA extraction, the Y2 (but not Y1) samples were thawed then heated at 37°C for 5 to 10 min to analyze volatiles present in the sample for a separate pilot study. The incorporation of this pilot study in Y2 necessitated DNA extraction to be performed at the home institution, Mayo Clinic, rather than UMNGC. For both Y1 and Y2 samples, the swab tips were cut and genomic DNA extraction was performed by using a DNeasy PowerSoil kit (Qiagen, Hilden, Germany). For further details on sequencing and bioinformatics processing, see the supplemental materials and methods.

Taxonomy at the genus level was assigned using the SILVA database (68). Further analyses of individual species and unidentified operational taxonomic units (OTUs) used the top NCBI BLAST (69) search result for species-level identification, unless the percent identity was <90%, in which case the species was considered unknown.

### Community and diversity metrics.

Clustering into community state types (CST) was determined by the most abundant species in a sample. Samples with Lactobacillus crispatus, L. iners, L. gasseri, or L. jensenii as the most abundant species were classified as type I, II, III, or V, respectively. Samples with any other species as the most abundant were classified as type IV. Long-term CST transitions were defined as a change from one CST (former) to another (latter) that persists for more than 10 days or until the end of the study if the transition began within the last 10 days of the study. Ten days was chosen as the cutoff to define long-term transitions as persisting beyond menses, which typically does not last longer than 10 days (70). The transition window was defined as the day the dominant species of the latter CST began to increase in relative abundance, until the first day the dominant species of the latter CST exceeded 50% relative abundance and remained the most abundant species for 10 or more days or until the end of the study if transition began within the last 10 days of the study.

Ecological diversity metrics alpha diversity and beta diversity were employed to characterize microbial composition. Alpha diversity was measured using the Shannon index (see the supplemental materials and methods [Text S1]). Beta diversity was calculated using Bray-Curtis dissimilarity. The community rate of change was calculated using log Jensen-Shannon rate of change, log(*D_JS_*/Δ*t*), as described previously (32), where *D_JS_* is the Jensen-Shannon divergence of consecutive samples and Δ*t* is the number of days between samples.

### Exclusion criteria.

Participants with fewer than 12 vaginal swab samples (*n *=* *10, 28% of total enrolled) were removed from the study, while those who submitted 12 or more samples were included in the study (*n *=* *26, including 4 repeat participants, 72% of total enrolled).

In addition to exclusions based on sample collection compliance, data were excluded from analyses of specific factors based on compliance with those factors (see Table S1 in the supplemental material). Participants who only reported menstruating on one or fewer sampling days were excluded from menstrual cycle analyses (remaining *n *=* *17, including 3 repeat participants). Days on which participants failed to report menstruation status were assumed to be “not menses,” unless the participant reported menses on both the previous and following days. In that case, those days were considered to be “menses.” For all menstrual cycle analyses that require normalization of cycle days, only individuals who reported 1 or more full menstrual cycles (reported to have begun menses twice during the study) were included (*n *=* *8). Because three participants were using unique contraceptives and therefore could have self-identified based on the type they were using, data from these three participants are not presented or discussed in the contraceptive analyses. For diet analyses, one participant who reported fewer than 3 complete days of diet entries was excluded. Among the remaining participants, days on which less than 500 total calories were reported were removed, as the low caloric count is likely due to missing information. For exercise analyses, all participants who reported exercise at least once (*n *=* *23) were examined. Only participants with more than 10 mood entries during the study were included in the mood analysis (*n *=* *5).

10.1128/mSphere.00593-20.10 TABLE S1 Exclusion criteria and resulting number of participants (*n*) for specific analyses. Download Table S1, DOCX file, 0.01 MB. Copyright © 2020 Song et al. 2020 Song et al. This content is distributed under the terms of the Creative Commons Attribution 4.0 International license.

### Calculation of daily nutrient intake.

Participants reported meals through the web application by selecting food from a drop-down menu based on the selected dining hall. Food items were linked to nutritional information provided by the college’s dining service provider, including total calories, sugar, fiber, protein, fat, trans fat, saturated fat, carbohydrates, cholesterol, and sodium. Daily nutrient intake was calculated as a proportion of daily caloric intake. For example, p_sugar = total daily sugar (g)/total daily calories (kcal). Manual entry of meals was also allowed. For the 1,066 food items that participants entered manually, the USDA food composition databases (https://ndb.nal.usda.gov/ndb/) were used to determine nutritional information. One serving size was assumed for each food item unless specified otherwise. Obscure food items that were not a close match to the database search results (*n *=* *134) were omitted. Dietary habits (vegetarian versus nonvegetarian) were determined by manually examining food entries for each participant.

### Mood ratings.

In year 2 of the study, participants were instructed to complete mood ratings each day on a scale ranging from 0 to 100. Participants were instructed to report their average mood for the entire day. “Happy or sad” was used to assess affect (“happy” is 100 and represents positive affect), and “relaxed or tense” was used to assess arousal (“relaxed” is 100 and represents low arousal). An “anxiety rating” was created as an approximation of negative affect and high arousal by multiplying “happy” and “relax” ratings, then using linear conversions to a scale of 0 to 100:

$$\text{Anxiety = 100 – }\frac{\text{(happy)(relax)}}{\text{100}}$$

### Statistical analysis.

Statistical analyses were conducted using R (v3.5.0). Where indicated, *P* values were corrected for false discovery rate using the Benjamini-Hochberg method (R function p.adjust, “BH” method). *P* values of <0.05 were considered significant.

### Cross-sectional comparisons.

For cross-sectional analyses comparing two groups, Wilcoxon signed rank or rank sum tests were performed. For comparison of *Lactobacillus* abundances between multiple groups of contraceptive use, one-way analysis of variance (ANOVA) with the year and contraceptive type as the predictors was used, followed by Tukey’s HSD. Linear regression was performed to determine associations between alpha diversity and exercise or specific nutrient intake. Stratified analysis with nonparametric covariable adjustment (R package “sanon”) (54) stratified by year was performed to assess differences in diversity between vegetarians and nonvegetarians while accounting for potential batch effects between years.

Beta diversity was determined using a permutational multivariate analysis of variance (PERMANOVA, R package “vegan”) using Bray-Curtis dissimilarity and 999 permutations stratified by subject. When screening for differences in individual species, only taxa present in at least one sample from more than 50% of participants were included.

### Binomial test for community state type transitions.

The probability that each participant exhibited a transition window covering the first day of menses, given a null hypothesis that CST transitions are not associated with menses or bleeding, was determined (see Text S1).

We note that one participant who transitioned exhibited low reporting compliance. For instance, in the third week of the study, this participant reported menses as a manual note entry, and menstruation status was not reported on any subsequent days. Therefore, the CST transition which occurred in the latter half of the study did not appear to occur during menses. However, if we assume that she follows a cycle between 19 and 33 days, she would have transitioned during menses. We deemed this assumption appropriate. If we do not make this assumption and consider four out of five participants to have met our criteria, our conclusions would remain (*P = *0.02 instead of *P* < 0.001).

### Longitudinal analysis of vaginal microbiota over the menstrual cycle.

Cycles were normalized to 28 days, with the start of menses as day 1, by multiplying the raw cycle day (*r*) on the *i*th day of the study for each participant *s* by the factor (28/*m*):

$$\begin{array}{l} n_{i,s}=r_{i,s}\left(\frac{28}{m_{s}}\right)\\ r_{i,s}=1\text{, . . . ,}m_{s} \end{array}$$

where *n* is the normalized cycle day and *m* is the raw length of the first reported full menstrual cycle. The Shannon index, log Jensen-Shannon rate of change, and *Lactobacillus* abundances were modeled over normalized cycle days using local regression (R function “loess,” span = 0.5).

The Fourier transform is a commonly used method to estimate the frequency spectrum of time-dependent data. The frequency spectrum allows for estimating the power and frequency at which periodic patterns occur. Least-squares spectral analysis (LSSA, R package “nlts”) (53) is an extension of the Fourier method to unevenly spaced data. LSSA was performed on Shannon index, log Jensen-Shannon rate of change, and *Lactobacillus* abundances over normalized cycle days. However, consecutive first days of menses were not reset to 1:

$$\begin{array}{l} n\prime_{i,s}=r\prime_{i,s}\left(\frac{28}{m_{s}}\right)\\ r\prime_{i,s}=1\text{, . . . ,}t_{s} \end{array}$$

where *t* is the total number of days participant *s* remained in the study. The inverse of the estimated frequency for a given peak was used to estimate the period (*T*).

### Data availability.

Sequencing files (FASTQ) are available through the National Center for Biotechnology Information Sequence Read Archive under BioProject PRJNA637322. Metadata are available upon request.
