# Supplementary material for: Daily Vaginal Microbiota Fluctuations Associated with Natural Hormonal Cycle, Contraceptives, Diet, and Exercise
Source: mSphere. 2020 Jul 8;5(4):e00593-20. doi: 10.1128/mSphere.00593-20 (PMC7343982; doi:10.1128/mSphere.00593-20)
Supplement: TEXT S1 [file mSphere.00593-20-s0001.docx]

**Supplemental Materials and Methods**

*Sequencing and Processing*

Both Year 1 (Y1) and Year 2 (Y2) samples underwent genomic DNA extraction using the DNeasy PowerSoil Kit (Qiagen, Hilden, Germany). However, instead of vortexing, TissueLyser (Qiagen, Hilden, Germany) for 20 minutes at 20 Hz was substituted for cell lysis for Y1 samples, or MP FastPrep (MP Biomedicals, Solon, OH) for 60 seconds at 6.0 m/s was used for Y2 samples. After extraction the DNA content was measured using PicoGreen (Thermo Fisher Scientific, Waltham, MA) for Y1 samples, or High Sensitivity Qubit (Life Technologies Corporation, Carlsbad, CA) on dsDNA broad range for Y2 samples. 16S rRNA sequencing targeting the V3-V5 hypervariable regions was done at UMNGC using a high-throughput next-generation Illumina (San Diego, CA) MiSeq sequencing platform (250 bp paired-end) for all samples. The forward primer was 5’TCGTCGGCAGCGTCAGATGTGTATAAGAGACAGCCTACGGGAGGCAGCAG3’, and the reverse primer was 5’GTCTCGTGGGCTCGGAGATGTGTATAAGAGACAGCCGTCAATTCMTTTRAGT3’.

Sequences were processed using the DADA2 software package (67) with the following parameters: “MAXEE” was set to 1.0, R1 read length cutoff was 260 bp, R2 read length cutoff was 210 bp. Chimeras were removed using the “consensus” method. Singleton OTUs and eukaryotic sequences were also filtered out. Average sequence reads per sample were 17,991 for Y1 samples (range: 1,551 to 37,350, *n* = 706) and 20,712 for Y2 samples (range: 1,024 to 37,516, *n* = 397), amounting to 20,924,310 sequence reads from 1103 samples after filtering. Because relative abundances, which are used for the rest of the analyses, are sensitive to low sequencing reads, samples with <1000 reads were removed (30 of 1146 total samples removed).

*Binomial test*

A binomial test was performed to determine the probability that all five participants with long-term transitions exhibited these community state type transitions covering the first day of menses. For each participant, given their reported days of bleeding and transition window length, we calculated the proportion of days in the study during which transition could have begun such that the transition window covers the first day of menses, to the total number of days in the study that the participant submitted samples. Using the probabilities *p* associated with each participant *s*, a binomial test on the observation that all five participants displayed transition windows covering the first day of bleeding was performed:

$$P(\mathbf{k}=\mathbf{x})=\prod_{s=1}^{n} (p_{s})^{x_{s}}(1-p_{s})^{(1-x_{s})}$$

where **k** is the vector of potential results (*k_1_*, …, *k_n_*) for *n* participants, and **x** is the vector of observed results (*x_1_*, …, *x_n_*) where *x_s_* is either 1 (participant *s* did transition during menses or bleeding) or 0 (participant *s* did not transition during menses or bleeding). Because all five participants transitioned during this time, **x** = (1, 1, 1, 1, 1).

*Community and diversity metrics*

The Shannon index was used to measure the alpha diversity of a microbial community by considering species richness and abundance:

Shannon index = $-\sum_{i=1}^{s} p_{i}\ln(p_{i})$

where *s* is the total number of OTUs, and *p_i_* is the relative abundance (n/N) of a particular OTU in a sample.
