# Supplementary material for: Daily Vaginal Microbiota Fluctuations Associated with Natural Hormonal Cycle, Contraceptives, Diet, and Exercise
Source: mSphere. 2020 Jul 8;5(4):e00593-20. doi: 10.1128/mSphere.00593-20 (PMC7343982; doi:10.1128/mSphere.00593-20)
Supplement: TABLE S1 [file mSphere.00593-20-st001.docx]

| Group | Year 1  *n* | Year 2  *n* | Total *n* | Inclusion Criteria | Corresponding Figure(s) |
| --- | --- | --- | --- | --- | --- |
| Enrolled | 20 | 16 | 36 (32 unique, 4 repeat) | None | None |
| Final Cohort | 14 | 12 | 26 (22 unique, 4 repeat) | 12 or more samples | Fig. 1, 2, S2 |
| Menstrual  Analysis (cross-sectional) | 10 | 7 | 17 (14 unique, 3 repeat) | Final cohort criteria & at least one reported menses | Fig. 3, S3 |
| Menstrual Analysis (longitudinal) | 4 | 4 | 8 (8 unique, 0 repeat) | Menstrual analysis (cross sectional) criteria & at least one full menstrual cycle | Fig. 4, S4, S5 |
| Contraceptives (cross-sectional) | 14 | 9 | 23 (20 unique, 3 repeat) | Final cohort criteria, excluding participants with self-identifiable contraceptives | Fig. 5 |
| Diet | 14 | 11 | 25 (21 unique, 4 repeat) | Final cohort criteria, excluding participant with less than 3 complete days of diet entries | Fig. 6a, S7 |
| Exercise | 13 | 10 | 23 (20 unique, 3 repeat) | Final cohort criteria & reported exercise at least once | Fig. 6b, S8 |
| Mood | 0 | 5 | 5 (5 unique, 0 repeat) | Final cohort criteria & more than 10 mood entries | Fig. S9, S10 |
